# The Design of a Controlled-Release Polymer of a Phytopharmaceutical Agent: A Study on the Release in Different PH Environments Using the Ultrafiltration Technique

**DOI:** 10.3390/polym16243492

**Published:** 2024-12-14

**Authors:** Oscar G. Marambio, Alejandro Muñoz, Rudy Martin-Trasancos, Julio Sánchez, Guadalupe del C. Pizarro

**Affiliations:** 1 Departamento de Química, Facultad de Ciencias Naturales, Matemáticas y Medio Ambiente, Universidad Tecnológica Metropolitana (UTEM), J. P. Alessandri 1242, Santiago 7800002, Chile; 2 Departamento de Química Física, Facultad de Química y de Farmacia, Pontificia Universidad Católica de Chile, Santiago 7820436, Chile; aamunoz13@uc.cl; 3 Departamento de Química de los Materiales, Facultad de Química y Biología, Universidad de Santiago de Chile (USACH), Santiago 9170002, Chile; rudy.martin@usach.cl; 4 Departamento de Química Orgánica, Facultad de Química y de Farmacia, Pontificia Universidad Católica de Chile, Santiago 7820436, Chile; julio.sanchez@uc.cl

**Keywords:** polymeric controlled release system, herbicide 2,4 D, ultrafiltration system

## Abstract

A series of hydrophilic copolymers were prepared using 2-hydroxyethyl methacrylate (HEMA) and itaconic acid (IA) from free radical polymerization at different feed monomer ratios using ammonium persulfate (APS) initiators in water at 70 °C. The herbicide 2,4-dichlorophenoxy acetic acid (2,4-D) was grafted to Poly(HEMA-*co*-IA) by a condensation reaction. The hydrolysis of the polymeric release system, Poly(HEMA-*co*-IA)-2,4-D, demonstrated that the release of the herbicide in an aqueous phase depends on the polymeric system’s pH value and hydrophilic character. In addition, the swelling behavior (Wt%) was studied at different pH values using Liquid-phase Polymer Retention (LPR) in an ultrafiltration system. The acid hydrolysis of the herbicide from the conjugates follows a first-order kinetic, showing higher kinetic constants as the pH increases. The base-catalyzed hydrolysis reaction of the herbicide follows a zero-order kinetic, where the basic medium acts as a catalyst, accelerating the release rate of the herbicide and showing higher kinetic constants as the pH increases. The differences in the release rates found for the hydrogel herbicide at different pH values can be correlated with the difference in their swelling capacity, where the release rate generally increases with an increase in the swelling capacity from water solution at higher pH values. The study of the release process revealed that all samples in distilled water at a pH of 10 are representative of agricultural systems. It showed first-order swelling kinetics and an absorption capacity that conforms to the parameters for hydrogels for agricultural applications, which supports their potential for these purposes.

## 1. Introduction

Synthetic polymers have the advantage of being abundant and of high purity. They can be used in various applications due to the varied functional groups in their chemical structure [1,2]. Copolymers can synergistically have the characteristics of two different monomers [3,4] to obtain copolymers of the hydrogel type, which are capable of responding to certain stimuli of variation in pH and temperature. However, controlling the polymers’ primary structures to improve their physicochemical properties is of the utmost importance for their application in adsorption studies [5].

In this context, a superabsorbent polymer (SAP) is a water-absorbing hydrophilic polymeric system that can retain enormous amounts of a liquid relative to its own mass [6]. Also, the wide variety of functional groups that can be constituted in hydrogels allows for efficient water adsorption through physical or chemical interactions [6]. Hydrogels can be classified into two groups depending on the nature of the cross-linking reaction. If the cross-linking reaction involves the formation of covalent bonds, then the hydrogels are termed permanent hydrogels. On the other hand, if the hydrogels are formed due to physical interactions, viz. molecular entanglement, ionic interaction, and hydrogen bonding, then the hydrogels are termed physical hydrogels or stimuli-responsive hydrogels. In conventional hydrogels, the equilibrium swelling does not change with the changes in the surrounding environment’s pH, temperature, or electric field [7]. In contrast, stimuli-responsive hydrogels are polymeric networks that change their equilibrium swelling with the change in the surrounding environment [7,8]. These materials are made up structurally of cross-linked polymeric networks, allowing them to respond to specific environmental stimuli [9,10]. Furthermore, it has been reported that hydrogels’ hydration and adsorption capacities depend on the medium’s pH, the osmotic pressure, and the ionic strength. [11,12]. Modifying these parameters in the working medium allows the hydration capacity of hydrogels and adsorbing species to be enhanced in the solution [13]. We have previously reported that acrylate-group-based hydrogels have shown excellent performance due to their high reactivity in free radical polymerization [4,14,15]. In recent years, stimuli-responsive hydrogels have become a hot topic. A variety of methods have been reported for obtaining such hydrogels, which are sensitive to the temperature and pH, as well as to chemical, optical, electrical, and other factors [10,16]. Smart hydrogels display a significant physiochemical change in response to small changes in the surroundings. However, such changes are reversible; therefore, the hydrogels can return to their initial state after a reaction, as soon as the trigger is removed [17]. Among the various types of stimuli-responsive materials, magnetic soft materials have shown remarkable progress in their design and fabrication, leading to the development of soft magnetic robots with unique advantages and potential for many important applications [12,17].

In the past decade, cellulose has been one of the most preferred compounds for the fabrication of hydrogels due to its natural origin, renewability, compatibility, and abundance and due to the direct use of native cellulose through physical cross-linking. For such applications, additional functional groups on the anhydroglucose unit of cellulose are often required to act as cross-linking sites, thus introducing additional chemical reagents into the system. An oxidation reaction is one of the most common ways to convert cellulose into a value-added derivative and obtain numerous functional groups (–COOH, –CHO) [18].

On the other hand, hydrogels have received increasing attention due to their significance and applications in areas such as the immobilization of enzymes, solute separations, baby diapers, soil for horticulture and agriculture, drug delivery, adsorbent pads, etc. [19,20,21,22,23,24,25,26,27,28,29,30,31]. Accordingly, one of the most important agricultural activities is the irrigation process, which facilitates the use of soil nutrients by plants and pest control, which helps crop growth. [32,33,34]. It has been reported that cross-linked polyacrylamide hydrogels, added to soils at rates of between 5 and 10 g/kg, reduce water infiltration into soils by up to 87–94% [32].

Furthermore, irrigation processes are costly in areas with water scarcity, so hydrogels have become an attractive alternative to solve this problem. This is even more reason for SAPs to stand out. They reduce the cost and provide the necessary continuous irrigation for this process [35]. It has been reported that these materials with granular structures also contribute to stabilizing the soil structure, improving the aeration, permeability, and soil quality, reducing soil compaction, and providing a favorable environment for crop growth [36].

Hydrophilic functional groups that are attached to the main chain of the polymer play a fundamental role in the hydration capacity, and its resistance to dissolution arises from three-dimensional cross-linked polymer networks that are produced from the simple reaction of one or more monomers [37,38]. They show a hydration capacity that retains a significant part of water or another fluid solvent in their structures, which generally do not dissolve [39].

Within the research area, an innovative cellulose-based SAP has been reported with absorption of 74 g of distilled water per g of SAP; however, when evaluating the absorption of tap water, this figure was 40 g, which is within the range of the typical absorption capacity of an SAP in a saline medium, which is 30–60 g water/g SAP. When subjected to extreme pHs, the system that was treated in that research showed a swelling that was close to the upper limit in the case of a basic pH and even exceeded it in an acidic medium [40]. In addition, other work has found that hydrogels swell better at neutral pHs, meaning that these results could even be superior in real agricultural systems [41].

Recently, polymers designed to support agricultural chemicals have been developed to address the serious environmental issues associated with traditional agrochemicals [42,43,44,45,46]. Controlled-release formulations for delivering herbicides offer both ecological and economic benefits [47]. Special attention is given to degradable polymeric materials and hydrogels, as they serve dual functions in this application [48]. One of the most commonly used monomers for synthesizing hydrogel materials is HEMA due to its hydroxyl functional group (OH-), which interacts with water molecules through strong hydrogen bond interactions that generate swelling [49,50]. A series of cross-linked polymers containing functional groups based on the acrylic acid (AA) monomers [39,51] N- vinylpyrrolidone (NVP) [37,49] and itaconic acid (IA) [52,53] have been described and possess a high hydration capacity due to their molecular structure, generating proper hydrophilicity. Controlled-release polymeric systems have been developed, in which bioactive compounds are covalently bonded to the polymer backbone. This approach helps reduce environmental contamination, a common issue associated with biologically active agents that release their compounds through hydrolytic or enzymatic cleavage of the bonds [53].

Herbicide 2,4-D is a phenoxy herbicide, which functions as a synthetic plant hormone. It effectively and selectively controls broadleaf weeds in cereal crops, without affecting legumes, as well as in corn crops. Additionally, 2,4-D is compatible with most pesticides that are commonly used in agriculture [54].

Systematic literature reviews on synthesizing synthetic copolymers and hydrogels are widely reported in this context. However, no records of herbicide-releasing synthetic polymers are based on HEMA and IA. The contribution of this work is in the design of a controlled-release polymer, 2,4-D, which is proposed as a model for other phytodrugs using hydrophilic polymers, which initially was obtained as a linear polymer through free radical polymerization in a solution with an organic solvent, an initiator, without a cross-linking agent. Subsequently, the SAP was obtained based on the functional groups that were activated through chemical modification in a basic environment to enhance the physical bonds through a hydrogen bridge between the chains; that is, it becomes a physical hydrogel later to be able to characterize its structure and incorporate the herbicide into the main chain through a condensation reaction. The novelty of this work is based on two important contributions: (1) The polymer was obtained as a linear polymer by free radical polymerization in a solution, using an organic solvent and initiator without a cross-linking agent. The functional groups of the hydrophilic monomers were activated by a chemical modification in a basic environment to enhance the hydrogen bridge bonds between the chains, giving rise to the SAP, which is a physical hydrogel, to characterize its structure later and incorporate the herbicide into the main chain through a condensation reaction. (2) The design of a controlled-release polymer of a phytopharmaceutical agent (herbicide) was implemented to quantify its release in different pH environments, with the purpose of adapting these materials to agricultural activities for improving soil quality, ensuring the efficient use of water in the agricultural sector with drought. The LPR technique (ultrafiltration system) was used to study the controlled release of the herbicide from the prepared system.

## 2. Experimental Procedure

### 2.1. Reagents

Hydroxyethyl methacrylate (HEMA) (Merck, Stuttgart, Darmstadt, Germany) was distilled under reduced pressure. Itaconic acid (IA) (Merck, Stuttgart) and the herbicide consisted of 2,4-dichlorophenoxy acetic acid (2,4-D), C_8_H_6_Cl_2_O_3_, m.p. 134–138 °C (Chemika, Fluka, Buchs, Switzerland), were used as they were obtained while all the other reagents and solvents were used as received. Ammonium persulfate (APS) was used as an initiator. All reagents were commercially obtained from Sigma-Aldrich (St. Louis, MO, USA), and no further purification was performed prior to the synthesis of copolymers.

### 2.2. Measurements

The Fourier transform infrared (FT-IR) spectrum was recorded using a PerkinElmer (Waltham, MA, USA) 2000 using the attenuated total reflection–FT-IR method at room temperature. The structure of the polymer and photoactive polymers were determined by proton nuclear magnetic resonance (^1^H NMR) on a Bruker 400 MHz spectrometer, Karlsruhe, Germany. Gel permeation chromatography (GPC) was performed to determine the number-average molecular weight (Mn) and weight-average molecular weight (Mw) under the following conditions: a WATERS 600E instrument equipped (Kioto, Japan) with UV and RI detectors, using THF as a solvent at a flow rate of 1.0 mL/min. The samples were analyzed at 30 °C with a concentration of 6 mg/mL, and calibration was conducted using poly (methyl methacrylate) (PMMA). The UV-VIS spectra were obtained using a Perkin-Elmer model Lambda3 spectrometer. LAB LONCO 6L equipment was used for continuous freeze-drying (lyophilization). The pH was measured using a Hanna 211 ThermoFisher pH meter (Dublin, Ireland). Subsequently, the LPR technique system was used to evaluate the controlled release of the bioactive agent 2,4-D from the polymeric matrix system. Details have been previously described [14,55].

### 2.3. Synthesis of the Copolymers

**Figure 1:**
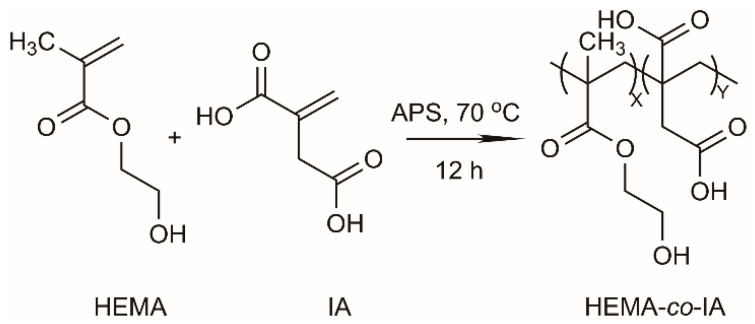
Synthetic route of the synthesis of the Poly(HEMA-co-IA) matrix.

P(HEMA-co-IA) was synthesized via radical copolymerization using different feed monomer ratios in an aqueous solution. A total of 0.5 mol% of APS was used as the initiator at 70 °C in 15 mL of bi-distilled water, maintaining a total monomer concentration of 12 mM [31]. The solution was purged with nitrogen gas (N_2_), and the copolymer systems were placed in a temperature-controlled bath at 70 °C for a copolymerization reaction time of 12 h. The polymer was then precipitated using heptane and dried under reduced pressure at 50 °C. The yield of the reaction was 85%. A schematic representation of the synthesis of the P(HEMA-co-IA) hydrogels is shown in Figure 1.

### 2.4. Activation of the Functional Groups of the Controlled-Release Polymer

**Figure 2:**
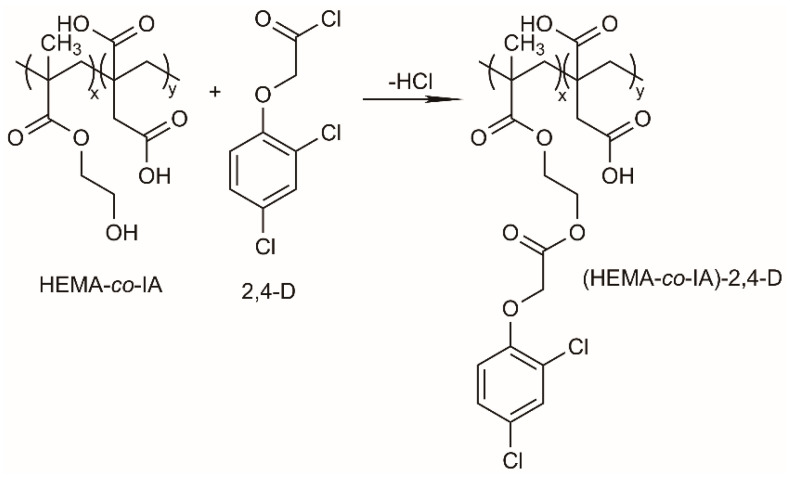
Synthetic route of the esterification reaction of the Poly (HEMA-co-IA) -2,4- conjugate.

The Poly(HEMA-co-IA) copolymer underwent chemical modification in a basic environment to enhance its swelling properties. The procedure involved dissolving 100 mg of the copolymer in 5 mL of distilled water and then increasing the pH to a highly basic level until the copolymer was visibly dissolved. This process formed a superabsorbent polymer (SAP) hydrogel, which was subsequently dried using freeze-drying techniques. The SAP hydrogel was created through physical interactions, specifically hydrogen bonding, among its functional groups. The hydrogel’s equilibrium swelling capacity varied with changes in the surrounding environment, particularly at different pH levels. Following this, the herbicide 2,4-D was covalently attached to the polymer chain through a condensation reaction, see Figure 2.

### 2.5. Esterification Reaction

The copolymer (300 mg) and the chloride (300 mg) were dissolved in 5 mL of dimethylformamide (DMF). This solution was transferred into a three-necked flask equipped with a nitrogen inlet and outlet, a dropping funnel, a magnetic stirrer, and a thermometer. While stirring, 1 mL of pyridine was added dropwise at approximately 0 °C. The reaction mixture was then heated to 25 °C for 1 h.

After the heating period, the solution was poured into a large volume of 0.5 M HCl solution to precipitate the product. The resultant product was filtered and washed several times with cold distilled water. To purify the product, a precipitation process using cold distilled water was conducted, followed by freeze-drying until a constant weight was achieved. The overall yield of the product was 70%.

### 2.6. Heterogeneous Hydrolysis of Polymeric Herbicides

**Figure 3:**
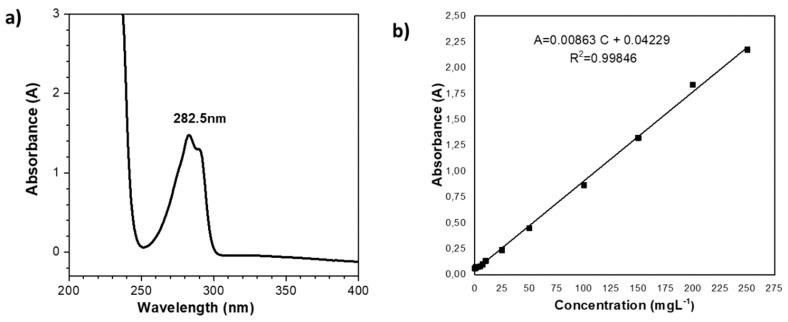
(**a**) UV-Vis spectrum of 2,4-D in CHCl_3_. (**b**) Calibration curve of herbicide 2,4-D.

The method described involved the retention of a polymer herb using a membrane filtration cell, followed by the separation of low-molar-mass species (bioactive molecules) from the copolymer matrix. Approximately 20 mg of Poly(HEMA-co-IA)-2,4D samples was mixed with 20 mL of water and placed into the membrane filtration cell while stirring. A membrane with an exclusion limit of 10,000 g/mol (AMICON PM 10 or equivalent) was used for this process. The total volume in the cell was maintained at 20 mL. The reservoir was filled with water that was adjusted to match the same pH levels (3, 5.4, and 10) as those in the cell solutions. The system was pressurized to 300 kPa, and the cell solution was stirred for 10 min. After this period, the system was washed with the reservoir fluid at a flow rate of 4–6 mL/min. Filtration fractions were collected daily for a duration of 10 days. The heterogeneous solution contained the released bioactive agent, and the concentration of this agent in the filtration fractions was quantitatively measured using UV spectroscopy at a wavelength of 282.5 nm, as shown in the UV-Vis spectrum (Figure 3a). A calibration curve for the herbicide 2,4-D was established (Figure 3b), utilizing an aqueous sodium hydroxide solution as the solvent. Tests were conducted at various pH levels of the reaction environment. Additionally, the copolymer–herbicide system was lyophilized for further analytical analysis.

## 3. Results and Discussion

### 3.1. Synthesis and Characterization of Poly(HEMA-co-IA)

**Figure 4:**
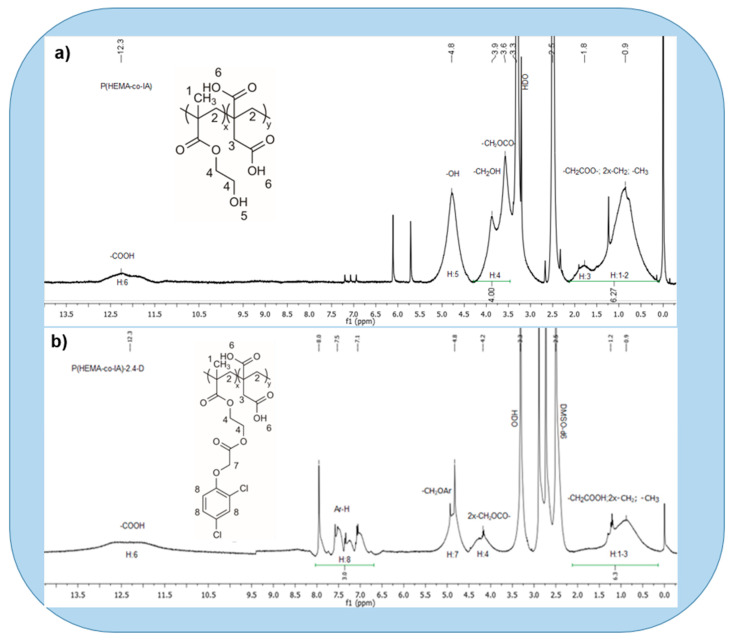
(**a**) ^1^H NMR spectrum of Poly (HEMA-co-IA) matrix and (**b**) Poly (HEMA-co-IA)-2,4D conjugate.

The copolymer poly(HEMA-co-IA) was synthesized through free radical polymerization in an aqueous solution. The reaction conditions and copolymer compositions are detailed in Table 1. This copolymer is soluble in DMF and dimethyl sulfoxide (DMSO). The number-average molecular weight (Mn) was found to be 18,700, while the weight-average molecular weight (Mw) was 23,500. The molecular weight distribution, measured by polydispersity (Ð = Mw/Mn), was calculated to be 1.32. The weight ratios of hydroxyethyl methacrylate and itaconic acid in the copolymer were determined using ^1^H NMR spectroscopy. The highest yield value corresponded to the copolymer at 75.5% for a composition of 76:24 mol.-% of the HEMA monomer, obtained at low conversions. The ^1^H-NMR (δ in ppm; DMSO-d6) spectrum of the copolymer exhibited the following signals: from 0.2 to 2.0 (9H, m broad, –CH_3_, 2x –CH_2_–, –CH_2_COOH aliphatic chain of HEMA and IA); at 2.8 [solvent DMSO-d6]; at 3.6 [–CH_2_ CH_2_OCO] from HEMA; at 3.9 (2H, s broad, –CH_2_OH, HEMA]; at 4.8 [–CH_2_CH_2_-OH from HEMA]; and at 12.3 [assigned to the-COOH from IA] [56] (see Figure 4a).

### 3.2. Copolymer Composition by ^1^H NMR

The relationship between the area of aliphatic protons from HEMA and IA monomers and the methylene protons from the HEMA unit was used to determine the copolymer composition, utilizing the integrals of the following functional groups: I_Met_ at 3.9 [2H, s broad, –CH_2_OH, HEMA]; and I_Alif_ from 2.0 to 0.2 [9H, m broad, –CH_3_, 2x –CH_2_–, –CH_2_COOH aliphatic chain of HEMA and IA]. To achieve this, we used three equations (Equations (S1)–(S3)). Applying these equations with I_Alif_ = 6.27 e I_Met_ = 4.0, the copolymer composition was obtained and is displayed in Table 1. The HEMA composition in the copolymer was 76.0%.

### 3.3. Poly(HEMA-co-IA) Matrix Grafted with 2,4 D-Chloride

The ^1^H NMR spectrum of the copolymer-2,4 D conjugate showed the following signals (δ in ppm; DMSO-d6): at 0.2–2.0 [9H, m, 2x –CH_2_, 1x –CH_3_; –CH_2_COOH] from the backbone of both monomer HEMA and IA units; at 2.8 [solvent DMSO-d6]; at 4.2 [4H, m, 2x–CH_2_OCO– from HEMA] and at 4.8 [2H, s, –CH_2_OAr; at 7.1 [1H, m, H_d_-Ar]; at 7.5 [1H, m, H_b_–Ar]; at 8.0 [1H, m, H_a_–Ar]; and at 12.3 [assigned to 1H, s, –COOH from IA], ref. [56] (see Figure 4b).

### 3.4. Degree of Functionalization of Poly(HEMA-co-IA)-2,4-D

The degree of functionalization was assessed using ^1^H NMR, which analyzed signals from functional groups that were characteristic of each monomeric unit. For this purpose, the integrals of the identified functional groups were used from 7.5 to 7.1 (1H, m, Hb–Ar; 1H, m, Hd–Ar) and from 2.2 to 0.2 (9H, m ancho, –CH_3_, 2x–CH_2_–, –CH_2_COOH, aliphatic chain copolymer), and subsequently, three equations were established (Equations (S4)–(S6)). Therefore, the copolymer by 2,4-D was functionalized approximately 100%, since the percentage of 2,4-D coincided with that of the HEMA in the copolymer.

**Figure 5:**
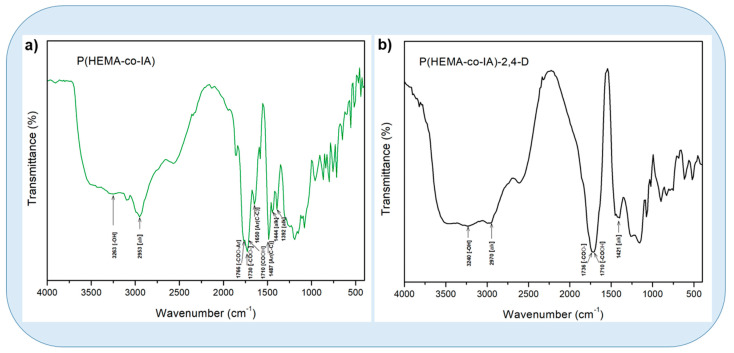
(**a**) Infrared spectra of P(HEMA-co-AI) matrix and (**b**) P(HEMA-co-AI) grafted with 2.4-D for a copolymer composition of 3.0:1.0.

The FT-IR spectra of the unmodified copolymer showed the presence of the following characteristic absorption bands: at 3240 ν [tension O–H; from HEMA, –COOH from IA]; at 2970 ν [tension C–H, –CH_2_–, CH_3_]; at 1736 ν [tension –C–O from –COO–, HEMA]; at 1710 ν [tension –C–O; –COOH, IA]; and at 1421 ν [bending C–H; –CH_3_, –CH_2_–] (see Figure 5a). On the other hand, the bands that were present in the FT-IR spectrum of the Poly (HEMA-co-IA) -2,4-D conjugate confirmed that the tension and bending vibrations corresponded (see Figure 5a).

The FT-IR spectra of the controlled-release system showed a significant change in the absorption band within the range at 3263 ν [tension O–H; –OH from HEMA and –COOH from IA]; at 2953 ν [tension C–H, –CH_2_–, CH_3_]; at 1776 ν [tension C–O; –COO–, Aromatic from 2,4-D]; at 1730 ν [tension C–O; –COO– from HEMA]; at 1710 ν [tension C–O, –COOH from IA]; and at 1650 and 1487 ν [bending C–H; –CH_3_, –CH_2_–] (see Figure 5b).

### 3.5. Determination of Monomer Reactivity Ratios

The values of the monomer reactivity ratios for HEMA and IA were determined from the ratios of the monomer feed and the composition of the copolymer obtained at high conversions. The Fineman–Ross [57], and Kelen–Tüdos [58] methods were used. The experimental data indicated random comonomer incorporation with a slightly (see Tables S1 and S2) alternating tendency. Figure S1 shows η−ξ plots according to the K-T method, from which the monomer reactivity ratios were determined for the copolymer. The variable ε can take any possible value within the 0 to 1 interval. A plot of η vs. ξ gives a straight line, which on extrapolation to ε = 0 and ε = 1 gives −r2/α and r1, respectively. The results included in Table S1 yielded reactivity ratios for HEMA and IA. The Kelen–Tudos method led to reactivity ratios for HEMA and IA of r_1_ = 1.081 and r_2_ = 1.098, respectively (see Figure S1a). The experimental data indicated that the system Poly(HEMA-co-IA) can be considered a random incorporation, with r1×r2=1.186.

The ε vs. *η* graph allows us to relate these variables through a linear equation and obtain the values of r1 and r2, as shown in Equation (1) (see Figure S1):

(1) η=2.2130·ε−1.1320

For ε=0;−r2/α=−1.1320, where α=0.9703; then, r2=1.098.

For ε=1;r1=2.2130−1.1320; then, r1=1.081.

The values of r1 (1.081) and r2 (1.098) are close to unity, indicating that the monomers are added in a statistically similar manner. This suggests that the monomers M1 and M2 exhibit a higher preference for incorporating the same type of monomer that is already present at the end of the chain. Consequently, both monomers tend to randomly integrate together, resulting in the formation of small segments consisting of the same type of monomer. The Fineman–Ross method yielded reactivity ratios for HEMA and IA of r_1_ corresponding to the slope of 1.1120 and r_2_ corresponding to the intercept of 1.1470, respectively.

Here, r1 × r2 = 1.2754. The graph of the initial composition (M1) vs. the copolymer composition (m1) shows that both comonomers tend to incorporate statistically at random (see Table S2 and Figure S1b), which was verified by the reactivity parameters obtained using the KT method (r1 = 1.081 and r2 = 1.098), which were both close to 1 (r1 and r2), agreeing with the values obtained by the FR method for copolymers at low conversions.

Here, X_0_ = M_1_/M_2_; *y* = m_1_/m_2_; M_1_ and M_2_ = Feed monomer ratio; m_1_/m_2_ = Copolymer composition.

G = X_0_/*y* (*y* − 1) (2)

F = X_0_^2^/*y* (3)

The G vs. F graph (see Figure S1b) allows one to correlate these variables through a linear equation and obtain the values of r1 and r2, according to the equation of F-R:

(4) G=1.1120 F−1.1470

where r1 corresponds to the slope of 1.1120, and r2 corresponds to the intercept of 1.1470.

This method can obtain the reactivity parameters for r1 = 1.1120 and r2 = 1.1470. The values obtained in the linear equation indicate that both values are close to 1 (r1 and r2), indicating that monomers M1 and M2 are added statistically and with the same frequency. The high reactivity of the monomers results in an alternating character in the copolymer. This observation applies to both the F-R and K-T methods.

### 3.6. Swelling Behavior of Hydrogel: Influence of pH

**Figure 6:**
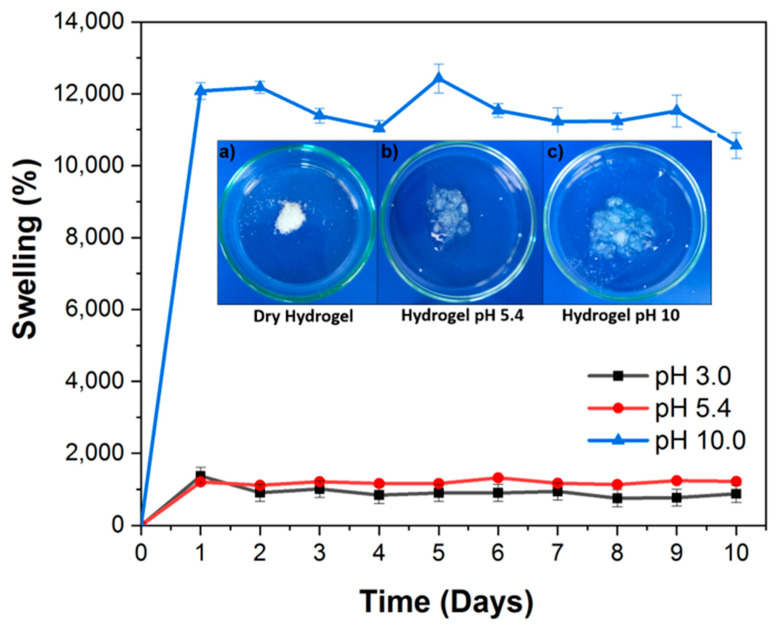
The swelling capacity of poly(HEMA-co-IA) hydrogel with a copolymer composition of 75:25 mol-% was studied as a function of pH in buffered solutions at pH values of 3, 5.4, and 10 at room temperature (25 °C). Inserted images (**a**–**c**) of the hydrogel dry, at pH 5.4 and 10.

The behavior of the poly(HEMA-co-IA) hydrogel, which has a copolymer composition of 75:25 mol-%, was investigated as a function of the pH. This was achieved by immersing the gels in buffered solutions at pH levels of 3, 5.4, and 10 at room temperature (25 °C). Figure 6a,b illustrate the swelling behavior of the poly(HEMA-co-IA) copolymers after various swelling times and at various pH levels. The hydration of the Poly (HEMA-co-IA) hydrogel at room temperature over a period of 10 days is presented as percentages of hydration (Sw %) based on the working pH in relation to the days of analysis. The copolymer’s dried samples were placed in a solution with a defined pH (3, 5.4, and 10) at 25 °C. Every hour, the sample was removed from the solution. After wiping off the water on the surfaces using moist filter papers, the hydrogels were weighed and recorded as Ws. The swelling ratio (Sw) was then calculated according to Equation (5):

(5) $$S_{W}(\%)=\frac{\left(W_{S}-W_{d}\right)}{W_{d}}\times 100$$

where W_s_ is the weight of the swollen hydrogel at an equilibrium state, and W_d_ is the weight of dried hydrogel (Xerogel). All the experiments were carried out in triplicate, and the average values were reported.

The swelling percentage of the hydrogel in acidic media (pH 3.0) reached equilibrium by day four, with swelling values varying from 970% to 913%. When the pH increased to 5.4, equilibrium was achieved within the first 24 h, with swelling values ranging from 1036% to 1221%. The maximum swelling equilibrium was reached within the first 24 h, with 10.445% and 12.375% values. These percentages were significantly higher than those observed at lower pH levels in acidic conditions. As a result, the hydrogel can achieve a weight gain of over 1200 times its original weight in pH 10 buffers. Additionally, the optimum absorption capacity for agricultural use was slightly surpassed, resulting in a weight increase of approximately 100 times. The complete dissociation of the acid groups in IA correlates with the dissociation constants pKa1 = 3.85 and pKa2 = 5.44 [59]. The swelling behavior of the hydrogel is influenced by the ionizable groups that are present in the polymer. These groups enhance the repulsive forces between the carboxylic groups and adjacent polymer chains. As a result, the swelling increases over time until it reaches an equilibrium point, known as the equilibrium swelling percentage.

When dealing with polymers that contain weakly ionizable groups, the absorption of the swelling medium is connected to the diffusion of mobile ions into the polymer. This process involves the ionization of fixed charges and the movement of the penetrant as the polymer continues to swell. Each monomer unit in the hydrogel carries an electrical charge, which aids in attracting and binding water molecules. As a result, each polymer molecule can retain a substantial amount of water [60].

The copolymer composition of 75:25 mol.-% has a significant impact on the equilibrium swelling behavior of the poly(HEMA-co-IA) gel at pH 10, as shown in Figure 6. Additionally, the increased presence of carboxylic acid from IA in the hydrogels enhances the formation of hydrogen bonds. This means that the swelling behavior of the hydrogels becomes more influenced by the IA content and the pH level.

### 3.7. Controlled-Release Hydrogels

The Poly(HEMA-*co*-IA) hydrogel with a copolymer composition of 75:25 mol-% was modified with a chloroacetylation reaction. The esterification reaction of HEMA as a comonomer was carried out using 2,4-D chloride. For this polymer-herbicide, it is suggested that the hydrolysis of the ester functional groups at different pHs facilitates the release of the herbicide. Therefore, a series of release tests were performed at various pH values. A sample of 20 mg in powder form was introduced into the ultrafiltration (UF) cell system using the LPR technique at pH levels of 3, 5.4, and 10, in accordance with the swelling studies. Filtration fractions (Z = 1–10) were collected daily for a duration of 10 days, starting with Z = 1 on the first day. The homogeneous solution contained released 2,4-D, quantitatively determined by UV spectroscopy at 282.5 nm using calibration curves.

In the washing and enrichment methods that utilize the LPR technique, the polymer-herbicide solution in the cell is described by Equation (6):

*R* = (*C_r_*/*C*0) × 100 (%) (6)

In this context, *C*r represents the herbicide concentration in the retentate, which is the cell solution volume ($V_{c}$) after processing a filtrate volume ($V_{f}$). *C*_0_ refers to the initial herbicide concentration in the cell. Additionally, the filtration factor (Z), expressed in relative units, is a useful characteristic of the process [61,62,63,64,65,66].

(7) $$Z=V_{f}\times V_{c}^{-1}$$

The irreversible reaction describes the polymer–herbicide (PL-herbicide) dissociation, which is described as follows:

$$PL-herbicide\overset{H^{+}/{OH}^{-}}{\to }PL+herbicide$$

Measurements taken before and after filtration determine the herbicide concentration that is not included in the polymer–herbicide.

**Figure 7:**
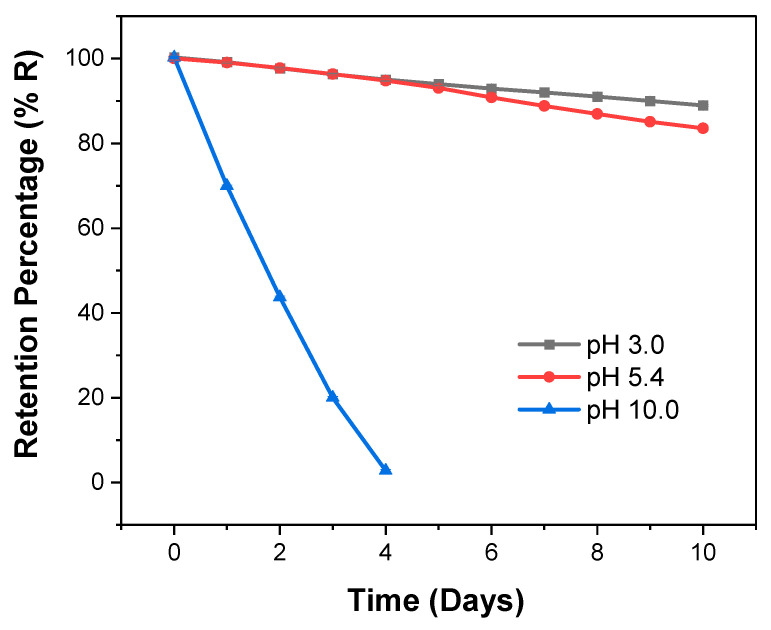
Release curves of herbicide using the P(HEMA-co-IA)-2,4-D hydrogel at different pH values.

The release profiles of the herbicide are illustrated in Figure 7. At pH values of 3.0 and 5.4, the retention capacities of the herbicide were 83.3% and 88.7%, respectively, after 10 days. In contrast, at a pH of 10.0, 97% of the herbicide was released by day four. Overall, the percentage of herbicide released from the copolymer increased at pH 10, with a significant release of the P(HEMA-co-IA)-herbicide observed at Z = 4 (four days). For example, at Z values of 1 and 2, the release rates ranged between 79% and 94.5%, with the highest release occurring at Z = 4. At pH 10, a significant release of the herbicide was observed after four days, reaching 97.3%. This high release rate is attributed to the hydrogels’ maximum absorption capacity at pH 10, which suggests that the acidic groups in the herbicide (IA) are completely dissociated. [59,60]. Ionizable groups within the polymer enhance the repulsive forces between the carboxylate groups and the surrounding chains. The swelling of the hydrogel increases over time but eventually reaches an equilibrium point, known as the equilibrium swelling percentage, after between 4 and 5 days. The dynamic absorption of the swelling medium occurs as the weakly ionizable groups attract and bind water molecules. This process significantly increases the volume of water within the hydrogel at a basic pH, which, in turn, accelerates the degradation of the polymer and leads to the release of the incorporated herbicide.

As the pH increases, the release of the herbicide also increases significantly. This is likely due to the effective hydrolysis of the ester groups at higher pH levels. The primary groups involved in this process are the hydroxyl groups from HEMA and the carboxylic acid groups from IA monomer units. At pH 10, the chemical equilibrium of the copolymer–herbicide conjugate shifts rapidly towards dissociation, facilitating their identification.

In contrast, at lower pH levels (3 and 5.4), the number of protonated hydroxyl and carboxylic acid groups along the polymer chains increases, thereby reducing the availability of free hydrophilic groups. This behavior at varying pH levels promotes the hydrolysis process at higher pH values. This phenomenon can be attributed to the weakening of the attractive forces that is caused by intramolecular hydrogen bonds within the chains at lower pH levels.

### 3.8. Release of Bioactive Agent: Kinetics of the Reaction

The washing method of the LPR technique involves filtering a low-molecular-weight species from the ultrafiltration system. When there is no interaction with the components of the ultrafiltration cell, including the ultrafiltration membrane, the instantaneous concentration of the species in the filtrate can be described by Equation (8):

(8) $$C^{filtrate}=C^{filtrate-init}e^{-F}$$

where *F* is the filtration factor, defined as $F=V^{filtrate}/V^{cell}$. The concentration of herbicide in the filtration fractions (<*c^filtrate^*>) is a mean value considering the instantaneous concentrations of the species in the collection process (*c^filtrate^*), which decrease during filtration.

If the kinetics of hydrolysis of the herbicide from the copolymer conjugates is in the order of one, there should be an exponential decay of *c^bound-day^* with time, according to Equations (7) and (8).

(9) $$-\frac{dc^{bound-day}}{dt}=kc^{bound-day}$$

and then

(10) $$c^{bound-day}=c^{bound-day-init}\exp(-kt)$$

where *k* is the kinetic constant.

The release of the bioactive agent from P(HEMA-co-IA)-2,4-D was studied at various pH levels at 25 °C. The results indicated that the highest levels of herbicide release occurred at pH 10, suggesting that basic hydrolysis plays a significant role in the release process. The analysis of the release mechanism showed that the samples with the best swelling characteristics at pH 10—representative of agricultural conditions—exhibited first-order swelling kinetics and an absorption capacity that is suitable for hydrogels used in agricultural applications. This supports their potential utility in such contexts. In contrast, lower levels of herbicide release were observed at pH values of 3 and 5.4, which aligns with the mechanism of hydrolytic breakdown of the herbicide–hydrogel bond. The release rates of the hydrogel conjugate at pH 3 and 5.4 are linked to their swelling capacity; a higher swelling generally increases the release rate from the water solution. The enhancement of conformational mobility can be influenced by either the extension of the lateral chain or by having sufficient –COO^–^ groups being hydrolyzed completely at pH 10 within the copolymer matrix. Furthermore, the extension of the lateral chain creates steric hindrance due to the HEMA moiety grafted with 2,4-D, which prevents the formation of inter- or intramolecular hydrogen bonding with the hydrophilic co-unit that is present in the IA segment.

The kinetic release constants (k) were determined for the system at pH levels of 3 and 5.4. These values were extracted from the slopes of the plots in Figure 7, as outlined in Equation (8). The results are presented in Table 2. The exponential decay observed in the release profiles indicates that the acid hydrolysis of the copolymer conjugate follows first-order kinetics when the pH shifts from 3.0 to 5.4. Additionally, the degradation rate of the polymer–drug linkage changes at a pH of 10. In contrast, the base-catalyzed hydrolysis of the herbicide exhibits zero-order kinetics, where the basic medium functions as a catalyst, enhancing the release rate of the herbicide and resulting in higher kinetic constants as the pH increases.

## 4. Conclusions

The characterization and application of a controlled-release polymer made from two hydrophilic monomers is reported. The polymeric controlled-release system, Poly(HEMA-co-IA)-2,4-D, was examined at different pH levels using the LPR technique. Based on the results of heterogeneous hydrolysis, we can conclude that the release rate of the herbicide depends on the pH of the reaction environment. Additionally, it was found that the acid-catalyzed hydrolysis of the herbicide follows first-order kinetics, with higher kinetic constant values being observed as the pH increases. In contrast, the base-catalyzed hydrolysis reaction follows zero-order kinetics, where the basic medium acts as a catalyst, accelerating the release rate of the herbicide and also showing higher kinetic constants with increasing pH levels. Consequently, the differences in release rates at various pH levels can be linked to variations in swelling capacity, with the release rate generally increasing as the swelling capacity rises under a basic pH. The release of the substance is positively influenced by the enhancement of conformational mobility, which can occur either through the extension of the lateral chain or by the complete hydrolysis of sufficient –COO– groups at a pH of 10 within the copolymer matrix. Furthermore, it can be concluded that the extension of the lateral chain leads to steric hindrance caused by the HEMA moiety, grafted with 2,4-D, which prevents the formation of inter- or intramolecular hydrogen bonding with the hydrophilic co-unit content from the IA segment.

Based on the results obtained, it is evident that the study of the release process demonstrated that all samples in distilled water at a pH of 10 are representative of agricultural systems. Additionally, the swelling kinetics exhibited first-order behavior, and the absorption capacity met the parameters for hydrogels used in agricultural applications, thereby supporting their potential for these purposes.

## Figures and Tables

**Table 1:** Experimental conditions, yield, and copolymer composition of Poly(HEMA-co-IA) hydrogels obtained using 15 mL of water at 70 °C and 12 h of reaction.

Monomer Feed Ratios HEMA/IA	HEMA mL (mmol)	IA g (mmol)	PSA mg (mol-%)	Yield %	Copolymer Composition by ^1^H NMR
3.0:1.0	4.51 (9.00)	1.59 (3.00)	53.6 (0.490)	37.7	3.0:1.0
2.5:1.0	4.30 (8.60)	1.80 (3.40)	54.1 (0.494)	37.3	2.5:1.0
2.0:1.0	4.01 (8.00)	2.10 (4.00)	54.4 (0.497)	32.4	2.0:1.0
1.5:1.0	3.61 (7.20)	2.53 (4.80)	54.5 (0.498)	27.1	1.5:1.0
1.0:1.0	3.01 (6.00)	3.16 (6.00)	54.5 (0.498)	28.5	1.0:1.0
1.0:1.5	2.41 (4.80)	3.79 (7.20)	55.9 (0.510)	46.5	1.0:1.5
1.0:2.0	2.01 (4.00)	4.22 (8.00)	56.0 (0.511)	42.0	1.0:2.0
1.0:2.5	1.72 (3.40)	5.51 (8.60)	56.4 (0.515)	38.6	1.0:2.5
1.0:3.0	1.51 (3.00)	4.75 (9.00)	55.4 (0.506)	30.0	1.0:3.0

HEMA: 2-Hydroxyethylmethacrylate; IA: itaconic acid; APS: ammonium persulfate.

**Table 2:** Rate constant (k) and half-life time (τ _1/2_) of the Poly(HEMA-*co*-IA)-2,4-D.

System	pH 3.0		pH 5.4		pH 10.0	
	k (n)	τ_1/2_ (days)	k (n)	τ_1/2_ (days)	k (n)	τ_1/2_ (days)
Poly(HEM -*co*- IA)-2,4-D	0.011 (0.988)	63.01	0.019 (0.981)	36.48	0.857 (0.424)	0.81

k: Rate constant; n: order of kinetic release.

## Data Availability

The original contributions presented in this study are included in the article/supplementary material. Further inquiries can be directed to the corresponding author.

## Supplementary Materials

The following supporting information can be downloaded at: https://www.mdpi.com/article/10.3390/polym16243492/s1, Figure S1: Reactivity ratios of Poly (HEMA-co-IA) by Kelen Tüdös method; Table S1: Reactivity ratios of Poly (HEMA-co-IA) by Kelen Tüdös method; Table S2: Reactivity ratios of Poly (HEMA-co-IA) by Kelen Tüdös method.
