# Supplementary material for: The Design of a Controlled-Release Polymer of a Phytopharmaceutical Agent: A Study on the Release in Different PH Environments Using the Ultrafiltration Technique
Source: Polymers (Basel). 2024 Dec 14;16(24):3492. doi: 10.3390/polym16243492 (PMC11728549; doi:10.3390/polym16243492)
Supplement: Supplementary file 1 [file polymers-16-03492-s001.zip › polymers-3366201-supplementary.pdf]

Supplementary Material

3.RESULTS AND DISCUSION

3.2 Copolymer composition by ^{1}H-NMR

The relation of the area corresponding to aliphatic protons from HEMA and IA monomers and methylene protons from HEMA unit were used for to determine the copolymer composition by ^{1}H-NMR, where the integrals of the following functional groups were used: at 3.9 (2H, s broad, -C**H**$_2$OH, HEMA); and from 2.0–0.2 (9H, m broad, -C**H**$_3$, 2x -C**H**$_2$-, -C**H**$_2$COOH aliphatic chain of HEMA and IA). and three equations were established, which are presented below:

Equation S1	$I_{Alif} = A(5X+4Y) = 6.27$	I_{Alif} = Integral aliphatic protons
Equation S2	$I_{Met} = A4X = 4.00$	I_{Met} = Integral methylene protons HEMA
Equation S3	$X+Y = 1$	X = Fraction X (HEMA)<br>Y = Fraction Y (IA)

Applying the equations with $I_{Alif} = 6.27$ e $I_{Met} = 4.0$, the copolymer composition is obtained as: X = 0.76; Y = 0.24. Thus, the calculation indicates that the HEMA composition in the copolymer is 76.0%.

3.4 Degree of functionalization of Poly(HEMA-co-IA)-*2,4-D*.

The degree of functionalization was determined by ^{1}H-NMR using signals from functional groups characteristic of each monomeric unit. For this purpose, the integrals of the following functional groups were taken from (1H, m, **H**b-Ar; 1H, m, **H**d-Ar); from 2.2–0.2 (9H, m ancho, -C**H**$_3$, 2x-C**H**$_2$-, -C**H**$_2$COOH, aliphatic chain copolymer) and subsequently three equations were established (**Equations S4–S6**), which are presented below:

Equation S4	$I_{Alif} = A(5X+4Y) = 6.3$	I_{Alif}= Integral aliphatic protons
Equation S5	$I_{Ar} = A3X = 3.00$	I_{Ar}= Integral aromatic protons
Equation S6	$X+Y = 1$	X = Fraction X (HEMA)<br>Y = Fraction Y (IA)

Applying the equations with IAlif = 6.3 and IAr = 3.0, the copolymer composition is obtained as: X = 0.76; Y = 0.24. Therefore, the functionalization was complete in the copolymer by 2,4-D is approximately 100%, since the percentage of 2,4-d coincides with that of HEMA in the copolymer.

3.5 Determination of monomer reactivity ratios

Table S1. Reactivity ratios of Poly (HEMA-co-IA) by Kelen Tüdös method (α = 0.9703).

Samples	(mol-%)	(mol-%)	Wt.-%	Xo	Y	*F*	ε	H
1	0.75	0.76	37.7	3.00	3.15	2.78	0.74	0.54
2	0.72	0.73	37.3	2.53	2.69	2.30	0.70	0.48
3	0.67	0.67	32.4	2.00	2.00	2.00	0.67	0.34
4	0.60	0.60	27.1	1.50	1.53	1.47	0.60	0.21
5	0.50	0.48	28.5	1.00	0.90	1.15	0.54	-0.05
6	0.40	0.40	46.5	0.67	0.66	0.68	0.41	-0.21
7	0.33	0.32	42.0	0.50	0.47	0.55	0.36	-0.37

8	0.28	0.28	38.6	0.40	0.39	0.41	0.29	-0.45
9	0.25	0.25	30.0	0.33	0.33	0.34	0.26	-0.52

The ε vs η graph allows us to relate these variables through a linear equation and obtain the values of r1 and r2, as shown in the equation 7 (see Figure **S1a)**:

Equation 1 $$\eta = 2.2130 \cdot \varepsilon - 1.1320$$

For $\varepsilon = 0;\ -r2/\alpha = -1.1320$, where $\alpha\ =\ 0.9703$, then $\ r2\ =\ 1.098$

For $\varepsilon\ =\ 1;\ r1\ =\ 2.2130 - 1.1320$, then $r1\ =\ 1.081$

The values of $r1$ (1.081) and $r2$ (1.098) are close to unity. Therefore, the monomers are added statistically, the monomers M1 and M2 tending towards a higher preference for the addition of a same type existing structural at the end chain, that is, both monomers tend to incorporate statistically at random, generating small segments of the same monomer.

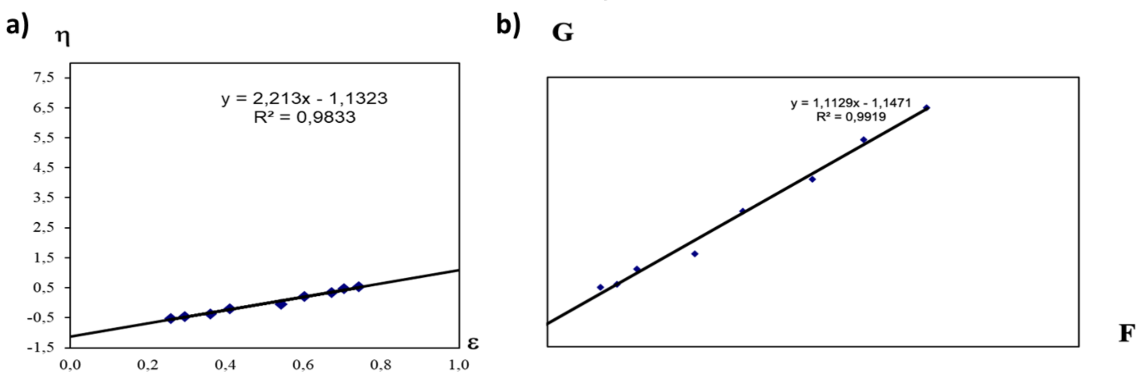

Figure **S1**. a) Graph of η vs ε, representation of the K-T parameters, b) Graph of G vs F, representation of the F-R parameters for the Poly (HEMA-co-IA) copolymer

The Fineman-Ross method (see Table **S1**) yielded reactivity ratios for HEMA and IA of r_1 corresponds to the slope 1.1120 and r_2 corresponds to the intercept 1.1470 respectively;

Where r1 x r2 = 1.2754. The graph of the initial composition (M1) vs the copolymer composition (m1) shows that both comonomers tend to incorporate statistically at random (see Table **S1** and Figure **S1b**), which is ratified by the reactivity parameters obtained by the KT method (r1 = 1.081 and r2 = 1.098) are both close to 1 (r1 and r2) and agrees with the values obtained by the FR method for copolymers at low conversions.

Table **S2**. Copolymerization reactivity ratios of poly (HEMA-co-IA) by Finemann-Ross method.

Samples	M1^a (mol-%)	m1 ^{c} (mol-%)	Xo	Y	[G]	[F]
1	0.75	0.76	3.000	3.149	2.047	2.858
2	0.72	0.73	2.529	2.690	1.589	2.378
3	0.67	0.67	2.000	2.003	1.002	1.997
4	0.60	0.60	1.500	1.525	0.517	1.475
5	0.50	0.48	1.000	0.905	-0.105	1.105
6	0.40	0.40	0.667	0.661	-0.342	0.672
7	0.33	0.32	0.500	0.473	-0.558	0.529
8	0.28	0.28	0.395	0.389	-0.621	0.402
9	0.25	0.25	0.333	0.330	-0.677	0.337

a) Monomer composition in feed, M_1 (mol-%); b) Monomer M_1= HEMA (M_2= IA)

c) Copolymer composition, m_1 in mol-%

Where: Xo is the molar ratio of HEMA and IA in feed; Y is the molar ratio of HEMA and IA in the copolymer.

The G vs F graph (see Figure 6) allows to correlate these variables through a linear equation and obtain the values of r1 and r2, according to the equation of F-R:

Equation 2 $$G = 1.1120\,F - 1.1470$$

Where r_1 corresponds to the slope 1.1120 and r_2 corresponds to the intercept 1.1470.

The reactivity parameters can be obtained by this method for r1 = 1.1120 and r2 = 1.1470, as results of the values obtained in the linear equation, both values are close to 1 (r1 and r2), then, indicating that monomers are added statistically, and with the same frequency both monomer M1 and M2.

The alternation is obtained due to the high reactivity that the monomers obtain when being in presence of water as solvent, obtaining a very close reactivity between both monomers and thus showing this alternating character in the copolymeric hydrogel. This applies to both the F-R and K-T methods.

3.8 Release of bioactive agent: Kinetic of the reaction

Mass balance:

$$\int_{F=0}^{F} c^{filtrate} dF = < c^{filtrate} > \Delta F \qquad \text{(S7a)}$$

Substituting (1) in (2) we obtain

$$\int_{F=0}^{F} c^{filtrate-init} \exp(-F) dF = < c^{filtrate} > \Delta F \qquad \text{(S7b)}$$

and integrating

$$c^{filtrate-init} = \frac{< c^{filtrate} > \Delta F}{1 - e^{-F}} \qquad \text{(S7c)}$$

As the filtration fraction obtained every day consists of 20 mL, so that final F = 1 and F = 1, it is found that

$$c^{free-day} = c^{filtrate-init} = \frac{< c^{filtrate} >}{1 - e^{-1}} \qquad \text{(S7d)}$$

Thus, $c^{free\text{-}day}$ corresponds to the concentration of the first differential volume obtained in the collection process ($c^{filtrate\text{-}init}$), and is calculated from the value of $<c^{filtrate}>$ corrected by Equation (S7e).

The concentration of herbicide bound to the polymer every day ($c^{bound\text{-}day}$) is given by

$$c^{bound-day} = c^{cell-day} - c^{free-day} = c^{cell-day} - \frac{< c^{filtrate} >}{1-e^{-1}} \qquad \text{(S7e)}$$

where $c^{cell\text{-}day}$ is the concentration of herbicide in the cell every day and is calculated following

$$c^{cell-day} = c^{cell-init} - \sum_{day} (< c^{filtrate} > \Delta F) \qquad \text{(S7f)}$$

where $c^{cell\text{-}init}$ is the initial concentration of herbicide in the cell.
